# Intensive alveolar recruitment after cardiac surgery: a randomized controlled clinical trial

**DOI:** 10.1186/cc13461

**Published:** 2014-03-17

**Authors:** A Leme, L Hajjar, M Amato, J Fukushima, C Hashizume, E Nozawa, E Osawa, R Nakamura, J Almeida, R Ianotti, J Auler, F Galas

**Affiliations:** 1 Heart Institute, São Paulo, Brazil; 2 Instituto do Cancer do Estado de São Paulo, Brazil

## Introduction

Protective mechanical ventilation has been associated with lower incidence of pulmonary and extrapulmonary complications in major surgery. The aim of the present study is evaluate whether adding an intensive alveolar recruitment protocol improves clinical outcomes and reduces healthcare utilization in patients undergoing cardiac surgery.

## Methods

In this single-center, parallel-group trial, we randomly assigned adult patients presenting signals of deficient gas exchange (PaO_2_/FIO_2 _<250 at a PEEP of 5 cmH_2_O) in the immediate postoperative period to either intensive alveolar recruitment or a standard protocol, both using low-tidal volume ventilation (6 ml/kg/ibw). Our hypothesis was that an aggressive alveolar recruitment protocol will be translated to better lung compliance, better gas exchange, fewer pulmonary complications and reduced length of hospital stay when compared with the control group.

## Results

A total of 320 patients were enrolled in the study, 163 patients in the standard protocol group and 157 in the intensive alveolar recruitment group. Patients of the interventional group presented a higher incidence of pneumonia than patients for the control group (5 (3.3%) vs. 19 (22%), *P *= 0.004). The length of the hospital stay was shorter among patients receiving intensive alveolar recruitment than among those receiving standard care (10.9 (9.9 to 11.9) vs. 12.4 days (11.3 to 13.6); *P *= 0.045). There was no difference between groups according to extrapulmonary complications and mortality.

## Conclusion

In this trial, an intensive alveolar recruitment protocol associated with a protective mechanical ventilation strategy reduced pulmonary complication and length of hospital stay in patients undergoing cardiac surgery (NCT01502332).
